# Supplementary material for: Splice-Junction-Based Mapping of Alternative Isoforms in the Human Proteome
Source: Cell Rep. Author manuscript; Available in PMC 2020 Jan 15. (PMC6961840; doi:10.1016/j.celrep.2019.11.026)
Supplement: 2 [file NIHMS1546469-supplement-2.zip › DF1/PXD010154/Liver/Liver_41_MINK1_AATAAGAGAAEAAGEGAAAAAGGHAGSAAGGGA.pdf]

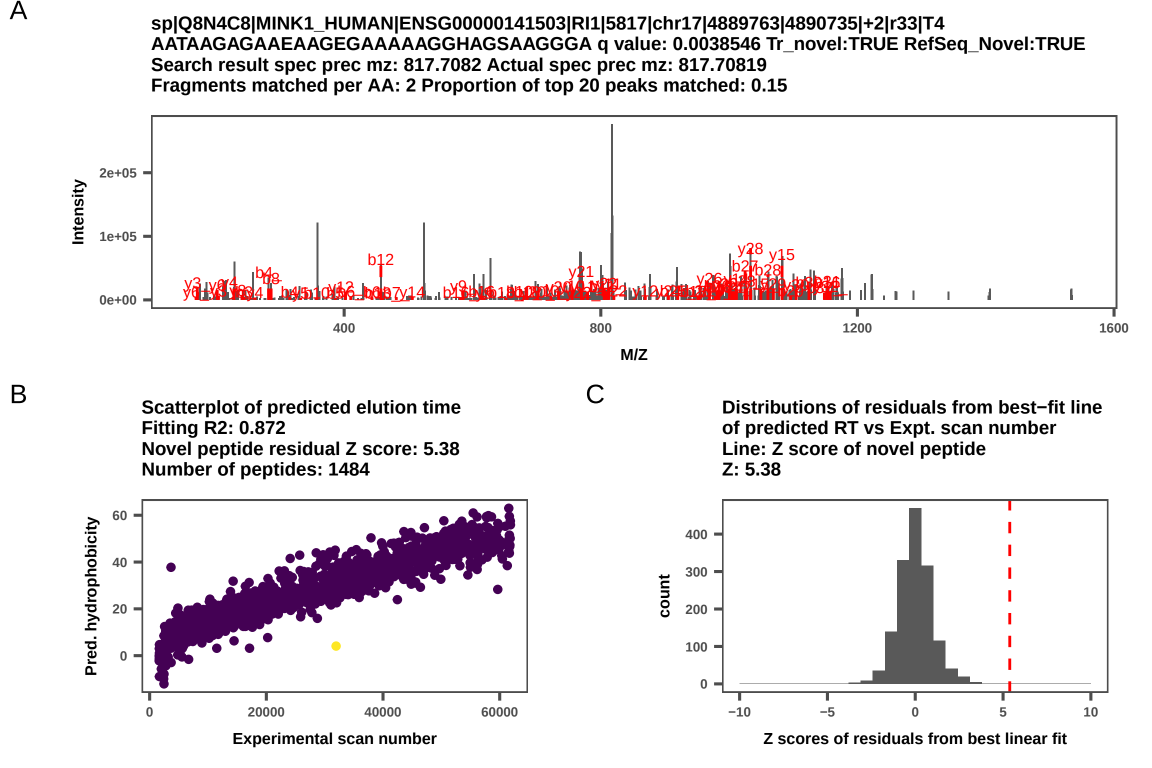
A
sp|Q8N4C8|MINK1_HUMAN|ENSG00000141503|RI1|5817|chr17|4889763|4890735|+2|r33|T4
AATAAGAGAAEAAGEGAAAAAGGHAGSAAGGGA q value: 0.0038546 Tr_novel:TRUE RefSeq_Novel:TRUE
Search result spec prec mz: 817.7082 Actual spec prec mz: 817.70819
Fragments matched per AA: 2 Proportion of top 20 peaks matched: 0.15
Intensity
2e+05
1e+05
0e+00
b12
y28
y15
y21
b27
b28
400
800
1200
1600
M/Z
B
Scatterplot of predicted elution time
Fitting R2: 0.872
Novel peptide residual Z score: 5.38
Number of peptides: 1484
Pred. hydrophobicity
60
40
20
0
0
20000
40000
60000
Experimental scan number
C
Distributions of residuals from best−fit line
of predicted RT vs Expt. scan number
Line: Z score of novel peptide
Z: 5.38
count
400
300
200
100
0
−10
−5
0
5
10
Z scores of residuals from best linear fit
